# Cytokines status in multiple sclerosis patients with comorbid recurrent depressive disorder

**DOI:** 10.1192/j.eurpsy.2021.1717

**Published:** 2021-08-13

**Authors:** E. Markova, I. Goldina

**Affiliations:** Neuroimmunology Lab, State Research Institute of Fundamental and Clinical Immunology, Novosibirsk, Russian Federation

**Keywords:** Multiple sclerosis, comorbid recurrent depressive disorder, Cytokines status

## Abstract

**Introduction:**

The presence of some common immunological pathogenesis mechanisms in multiple sclerosis and depression suggests the possibility of comorbid depressive disorder formation in multiple sclerosis patients, which significantly worsens their quality of life and patient’s compliance. In this regard, the depressive pathology diagnosis in people suffering from multiple sclerosis acquires important scientific and practical value.

**Objectives:**

The aim of the study was the cytokine status peculiarities identification in multiple sclerosis patients with comorbid recurrent depressive disorder (F33).

**Methods:**

The cytokines content in patient’s blood mononuclear cells culture supernatants was carried out by ELISA. The recurrent depressive disorder diagnosis was established based on ICD-10 criteria. The depressive disorders symptoms severity was determined according to the M. Hamilton and A.T. Beck depression scales, as well as during the clinical interview

**Results:**

A higher production of IL-6 was noted in multiple sclerosis patients with mild recurrent depressive disorder (F33.00), in contrast to patients without the affective symptoms. The IL-1β, TNF-α, IL-6 contents in the blood mononuclear cells culture supernatants of patients with severe recurrent depressive disorder (F33.2) exceeded the corresponding parameters of patients with mild depressive symptoms. A direct correlation between the depression severity and IL-1β, TNF-α, IL -6 spontaneous production by blood mononuclear cells of patients with multiple sclerosis was found.

**Conclusions:**

The severity of recurrent depressive disorder correlates with a change in the parameters of the cytokine status: severe depressive symptoms are accompanied by a change in the functional activity of immune cells and an increase in the production of cytokines synthesized by type I T-helpers.

**Disclosure:**

No significant relationships.

